# Paramedics’ Perceptions and Experiences Providing End-of-Life Care: A Cross-Sectional Survey

**DOI:** 10.3390/healthcare14193309

**Published:** 2026-10-04

**Authors:** Felicity Hasson, Natasha Campling, Sinead Keeney, Paul Slater, Sonja McIlfatrick, Julia Wolfe

**Affiliations:** 1 Institute of Nursing Research, Ulster University, Belfast BT15 1AP, UK; f.hasson@ulster.ac.uk (F.H.); pf.slater@ulster.ac.uk (P.S.); 2 Faculty of Health Sciences, University of Southampton, Southampton SO16 6YD, UK; n.c.campling@soton.ac.uk; 3 Doctoral College, Ulster University, 2-24 York Street, Belfast BT15 1AP, UK; sjmcilfatrick@ulster.ac.uk; 4 Northern Ireland Ambulance Service Health and Social Care Trust, Belfast BT8 8SG, UK; julia.wolfe@nias.hscni.net

**Keywords:** palliative care, delivery of service, paramedics, end-of-life

## Abstract

**Highlights:**

**What are the main findings?**

- There has been limited empirical work undertaken internationally in this area and much of the work does not explore how paramedics experience the challenges they face in practice in relation to end-of-life care. This paper provides a foundation for comparative research providing additional data that mirrors national and international themes, reinforcing the broader evidence with up-to-date data and findings.
- The paper highlights the challenges in the pre-hospital setting including limited access to patients’ records, medicines, and advice from other healthcare professionals, adding further to the evidence base.

**What are the implications of the findings?**

- Improved integration of information systems may support paramedics’ access to relevant Advance Care Planning documentation and treatment information at the point of care. Embedding palliative care competencies into paramedic training programmes and continuing professional development may help to build knowledge and confidence in providing end-of-life care.
- Accessible specialist palliative care advice and streamlined referral pathways may provide additional support for paramedics making decisions about end-of-life care and conveyance in the community.

**Abstract:**

**Background:** Many people spend their last year of life at home, with most wishing to die there. Evidence suggests paramedics facilitate over half of Emergency Department visits for palliative care patients, many of which are avoidable. **Objective:** To explore the perceptions and experiences of providing end-of-life care amongst paramedics in one region of the United Kingdom. **Design:** Online descriptive cross-sectional survey design using the ParAid Questionnaire. Quantitative data analysed using descriptive statistics. **Setting and Participants:** 182 paramedic respondents completed the questionnaire online (34% response rate). **Results:** Respondents reported numerous barriers to delivering end-of-life care. Access to medicines was problematic, with 54% reporting that they ‘always’ or ‘often’ had no access to specific medicines needed for symptom control. Education gaps were also evident: 67% reported that lack of pre-registration end-of-life care education and 71% that lack of post-registration education ‘always’ or ‘often’ influenced their ability to meet patients’ needs. Only 28% felt ‘very’ or ‘extremely’ competent in supporting patients and families in the last year of life, while 47% reported experiencing a ‘fear of doing the wrong thing’ ‘always’ or ‘often’. Access to information and referral pathways also presented challenges: 88% reported that they ‘always’ or ‘often’ lacked access to existing Advance Care Planning documentation, while 62% reported that they ‘rarely’ or ‘never’ had access to a specialist palliative care team out-of-hours. **Conclusions:** Findings emphasise the need for improved palliative care education, support structures, and improved access to information and medicines for paramedics. Findings identify potential areas for service development, including training, communication, and referral pathways, which may better support paramedics in providing end-of-life care.

## 1. Introduction

Many people spend the last year of their life at home, with most wishing to die there [1]. As patients progress towards end-of-life, they can experience worsening symptoms out-of-hours, and ambulance services are often contacted [2,3]. End-of-life care refers to caring for people likely to die within a year and is intended to enable people to live as well as possible until they die [4]. Internationally, evidence suggests paramedics facilitate over half of Emergency Department visits for patients receiving palliative care [5], many of which are avoidable [6]. Rates of Emergency Department attendance increase rapidly during the final three months of life with out-of-hours visits increasing more than in-hours visits [7]. Northern Ireland is projected to have the biggest rise in demand for palliative care across the United Kingdom, a 32% increase between 2023 and 2048 [8]. Lack of awareness of, or access to, palliative care services is a contributing factor leading to patients/families contacting ambulance services [6]. Responding to these calls can be challenging for paramedics for numerous reasons [2,6,9,10].

## 2. Background

The role of paramedics in providing end-of-life care has gained increasing attention as health systems seek to support patient wishes to die at home. There is limited empirical work undertaken internationally in this area and limited exploration of how paramedics experience challenges faced in practice. A recent global integrative review highlighted growing expectations for paramedics to deliver palliative care [6]. The review identified barriers, including limited access to patient records, insufficient training, and organisational culture oriented around life-saving rather than palliative care practice. It concluded that paramedics are a skilled workforce capable of facilitating home deaths and reducing hospital admissions but that a multi-faceted approach involving training, policy changes, and integration with multidisciplinary teams is needed to optimise their role in palliative care. 

In the United Kingdom, these findings are echoed in Campling et al.’s study, the first national survey of paramedic practice and experience providing end-of-life care [11]. Findings revealed paramedics frequently attended end-of-life patients, often without prior knowledge of their status or access to patient information, Advance Care Planning documents, or necessary medicines. Paramedics faced difficulties in accessing healthcare professionals for advice, especially out-of-hours, and reported limited pre- and post-registration training in palliative care, affecting confidence and competence. The study concluded that paramedics in England face multiple challenges in delivering end-of-life care, impacting quality of care, and increasing likelihood of hospital conveyance. The study emphasised the need for better integration of paramedics within the wider healthcare team, improved access to anticipatory medicines, 24/7 palliative care advice lines, and robust education and training programmes.

A recent international study demonstrated that this is not solely a United Kingdom issue [12]. Findings show that international Emergency Medical Systems report similar gaps in end-of-life preparedness, particularly communication, training, and cultural perceptions of death. Emergency Medical Services provide immediate care to those in need during medical emergencies [13]. In 2021, a scoping review mapped research on the role of Emergency Medical Services globally in providing palliative care to patients at end-of-life. Findings highlighted the emotional and ethical tensions faced by paramedics caring for patients who are actively dying and highlighted the ‘clash’ between emergency treatment and palliative care reality [14]. A more recent scoping review concluded that Emergency Medical Services play a vital role in end-of-life care, but face challenges such as limited education, health service barriers, and legal confusion over medicines. Integration between Emergency Medical Services and palliative care systems was recommended to improve patient autonomy, quality of life, and reduce healthcare costs [15].

There is emerging international evidence of attempts to formalise the paramedic role in end-of-life care. In Australia, researchers proposed a Delphi-informed framework for ‘palliative paramedicine,’ advocating for clearly defined competencies in symptom assessment, psychosocial support, and shared decision-making [16]. The study developed a comprehensive framework to standardise palliative paramedicine in Australia, with potential for international adaptation. 

### Rationale

Paramedic practice has changed significantly over the last 20 years, with greater professional autonomy, increasingly complex care delivered in community settings, and the introduction of degree level qualifications [2]. Northern Ireland provides an important context in which to examine the paramedic contribution to end-of-life care. Unlike other parts of the United Kingdom, health and social care is delivered through an integrated Health and Social Care system. The Northern Ireland Ambulance Service is the only regional ambulance service provider with a fully combined health and social care system in the UK. This requires paramedics to work across diverse geographical and service contexts and to interface with primary, community, hospital and specialist palliative care services when making decisions about care and hospital conveyance.

The need for palliative care in Northern Ireland is also projected to increase substantially. The region is predicted to experience the largest proportional increase in palliative care need across the United Kingdom, increasing by approximately 32% between 2023 and 2048 [8], alongside substantial growth in the oldest age groups. These demographic changes are likely to increase both the volume and complexity of end-of-life care required in community settings and as a result, the contribution of the ambulance service.

Despite these distinctive organisational and demographic characteristics, little is known about Northern Ireland Ambulance Service paramedics’ views of current end-of-life care practice, the challenges they encounter, or the support required to enable care at home. This study provides an opportunity to determine whether challenges identified in England and internationally are also evident within a regionally organised, integrated health and social care system, while identifying issues of particular relevance to local service development [11,17]. This survey aimed to explore paramedics’ perceptions and experiences of current end-of-life care practice and to identify factors perceived by paramedics to influence their ability to provide end-of-life care, alongside strategies to enhance the paramedic contribution to end-of-life care.

For the purposes of this study, end-of-life refers to the last 12 months of life, consistent with NHS England guidance [18]. The ParAid Questionnaire operationalises this timeframe using the term ‘last year of life’ when asking paramedics about their practice and experiences. This terminology provides a consistent timeframe rather than relying on individual interpretations of the term ‘end-of-life’. Accordingly, ‘last year of life’ is retained when reporting individual questionnaire items, while ‘end-of-life’ is used more broadly throughout the manuscript to refer to this 12-month period.

## 3. Methods

Online cross-sectional survey design was done using the ParAid Questionnaire [11]. Checklist for Reporting Results of Internet E-Surveys (CHERRIES) was followed [19]. ParAid questionnaire was adapted for use in a different region of the UK with permission and only minor modifications made including minor rewording and removal of response options that did not apply. Ethical approval was gained from Ulster University and Health Research Authority (IRAS 354259).

### 3.1. Sampling and Recruitment

Inclusion criteria required participants to be registered paramedics currently employed by the Northern Ireland Ambulance Service. Paramedics working in other healthcare environments were excluded. A census of all Northern Ireland Ambulance Service staff who met the inclusion criteria was conducted, resulting in the identification of 535 eligible paramedics. All 535 eligible paramedics were invited to participate in the survey. Four Clinical Pathways Leads and two Research Paramedics assisted with recruitment. Information about the survey and the survey link was emailed to paramedics via work email addresses, distributed through daily communications, displayed on posters in work areas and reminders were sent every other week. Participation was voluntary. The survey link was open for 8 weeks. Incentive in the form of a prize draw was offered.

### 3.2. Questionnaire Pre-Testing

The ParAid questionnaire was originally developed iteratively using evidence from the published literature, unpublished reports and policies, practitioner feedback, and Patient and Public Involvement and Advisory group review, and was subsequently piloted with paramedics for clarity of meaning and appropriateness to the survey objectives [11]. The questionnaire includes items addressing paramedics’ experiences and practices in end-of-life care, including frequency of attendance, awareness of end-of-life status, actions undertaken, access to medicines and patient information, conveyance and referral pathways, confidence and perceived competence, challenges to care, and education and training.

For use in the present study, the questionnaire was adapted for the Northern Ireland context with permission from Campling et al. [11]. Changes were limited to minor wording changes and the removal of response options that were not applicable within the Northern Ireland healthcare context.

The questionnaire was initially reviewed by eight other professionals including paramedics, academics and experts in palliative care for content and usability. The adapted questionnaire was pre-tested with four paramedics in Northern Ireland to assess the clarity and readability of the wording, understanding and interpretation of questions and response options, and the appropriateness of the adapted items. Feedback from the pre-test participants indicated that the questions and response options were clear, understandable and appropriate to the intended context, and no difficulties requiring further amendment were identified. No additional changes were made following pre-testing. Pre-test responses were not included in the study findings.

Internal consistency was assessed following the adaptation of the questionnaire. Cronbach’s alpha coefficients were calculated for relevant domains and were within acceptable ranges (0.725–0.895), supporting the reliability of the instrument in this population.

### 3.3. Data Collection and Analysis

The questionnaire was hosted on the JISC online survey platform. Completion took approximately 15 min. Data were collected from 4 August to 26 September 2025. All responses were anonymous. Quantitative data were analysed descriptively using the Statistical Package for Social Scientists (v29). Three members of the research team (SK, PS and FH) engaged in data analysis. A total of 182 respondents submitted the questionnaire. Percentages were calculated using the total study sample (n = 182) as the denominator. Individual questionnaire items could be left unanswered; therefore, item-level missing responses were retained in the denominator and are reported as ‘No response’ in the relevant tables.

## 4. Results

A total of 182 respondents submitted the questionnaire, representing a response rate of 34%. Although questionnaires were submitted by all 182 respondents included in the analysis, some individual items were unanswered; these item-level missing responses are reported as ‘No response’ in the relevant tables. Table 1 outlines the characteristics of the sample. To assess representativeness and potential selection bias, respondent demographics were compared with available 2025 Northern Ireland Ambulance Service workforce data. The age distribution of respondents was broadly comparable to that of the wider Northern Ireland Ambulance Service workforce across all age categories. Some variation was observed in role representation, with frontline clinical operations paramedics, specialist/advanced operational staff, and leadership staff proportionally more represented within the respondent sample. However, the survey directs respondents to ‘tick all that apply’ in relation to roles, whereas demographic workforce data classifies staff according to a single primary role designation. Direct comparison between role categories should therefore be interpreted with caution.

### 4.1. End-of-Life Patient Attendance

**Figure 1:**
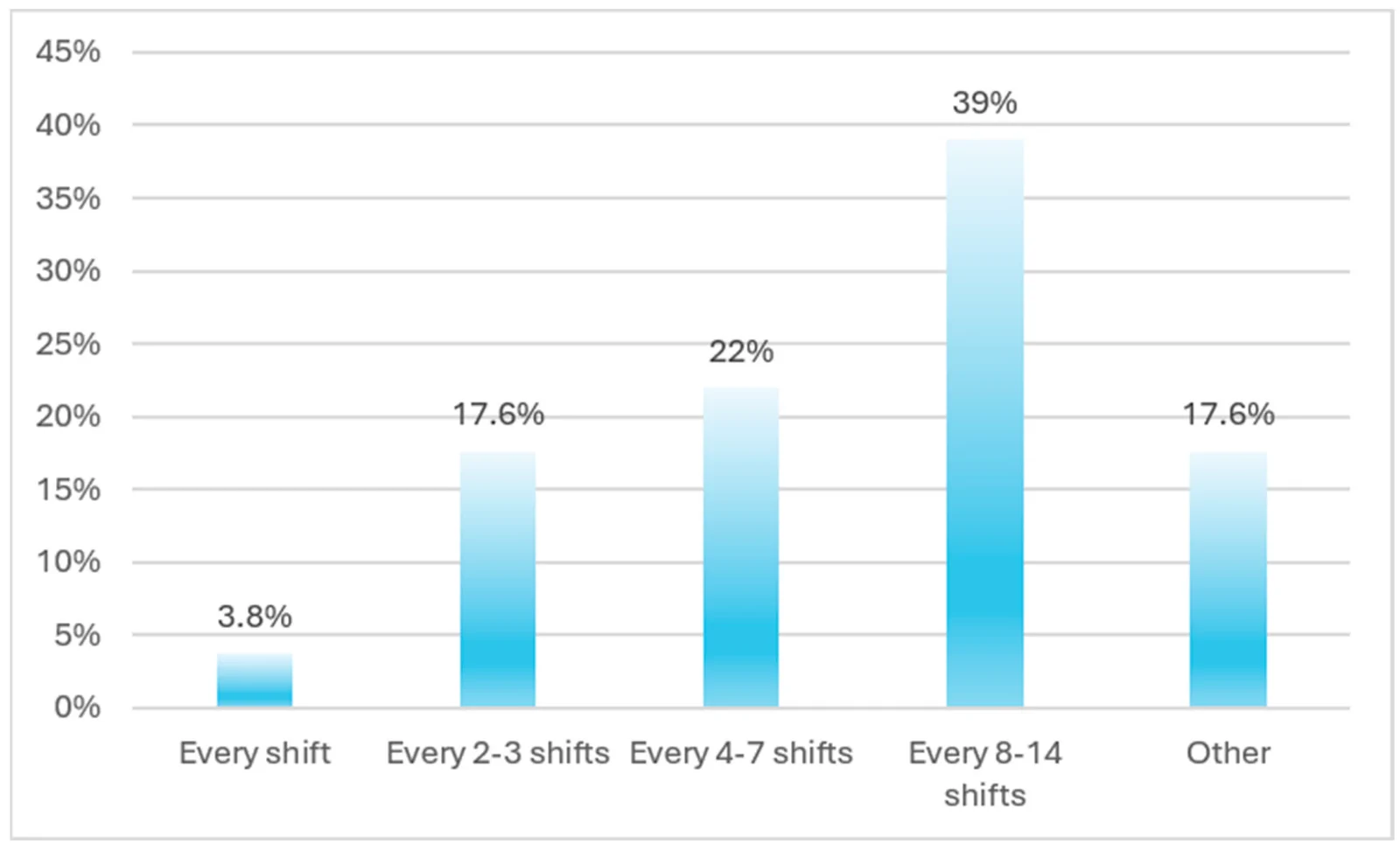
Frequency of Attendance with Patients in last year of life. Note: ‘Other’ was chosen by respondents who further explained that they felt they rarely attended patients in their last year of life.

Figure 1 shows respondents’ estimated frequency of attending patients considered to be in the last year of life, expressed in relation to the number of shifts worked rather than calendar days or weeks. Thirty-nine percent (n = 71) estimated that they attended such a patient once every 8–14 shifts worked, while 22% (n = 40) reported doing so once every 4–7 shifts worked. A further 18% reported attendance every 2–3 shifts and 4% every shift (Figure 1). As individual working patterns were not examined, these categories should not be interpreted as equivalent to specific day or weekly intervals.

### 4.2. Awareness of Patients at End-of-Life

Respondents reported limited prior awareness that a patient was approaching end-of-life before attending a call. Fifty-eight percent (n = 106) of respondents indicated that they ‘rarely’ or ‘never’ knew in advance that they were attending an end-of-life call, 34% (n = 61) indicated that they ‘sometimes’ knew and 8% (n = 14) ‘often’ or ‘always’ knew. Where end-of-life status was not known before arrival, information sometimes became available once paramedics were on scene. Thirty-nine percent (n = 70) of respondents reported that this ‘sometimes’ occurred, 22% (n = 41) reported that the information ‘always’ or ‘often’ became available in the form of Advance Care Planning documents or information provided by family/carers or patients. Despite limited prior information, respondents (70%, n = 127) indicated that they ‘always’ or ‘often’ suspected that a patient was within the last year of life, even if information was not recorded, 24% (n = 44) ‘sometimes’ suspected this and 5% (n = 10) stated that they ‘rarely’ or ‘never’ suspected this.

### 4.3. Frequency of Actions

Table 2 summarises the actions of paramedics when attending patients at end-of-life. Providing support and advice was commonly reported, with almost 90% (n = 163) indicating that they ‘always’ or ‘often’ provided support or advice to family and friends and over 60% (n = 110) reporting this frequency when supporting care or nursing home staff. Responses varied for clinical management and onward care. Almost forty-eight percent (n = 86) reported that they ‘sometimes’ administered medicines for symptom control. Similar percentages indicated that they ‘sometimes’ conveyed the patient to hospital (45.6%, n = 83) and 42.3% (n = 77) ‘always’ or ‘often’ did. Overall, 57.1% (n = 104) ‘always’ or ‘often’ referred to other healthcare providers for ongoing care. Almost 44% (n = 80) ‘rarely’ or ‘never’ made support arrangements for care after death; 25.3% (n = 46) ‘sometimes’ did; and 29.1% (n = 53) ‘always’ or ‘often’ did.

### 4.4. Access and Administering Medicines for Symptom Control

Respondents reported varying experiences regarding access to medicines required for symptom control at end of life. Over half, (54%, n = 99) indicated that they ‘always’ or ‘often’ had no access to specific medicines needed; 31% (n = 56) ‘sometimes’ and 15% (n = 27) ‘rarely’ or ‘never’. Respondents also reported on the availability of patients’ own just-in-case (anticipatory) medicines. Fifteen percent (n = 26) indicated that medicines were supplied from patients’ own anticipatory medicine supplies ‘always’ or ‘often’, while 34% (n = 61) reported this ‘sometimes’ and 48% (n = 88) ‘rarely’ or ‘never’. In relation to the authorisation to administer medicines, respondents indicated that they were authorised by patient group directions ‘always’ or ‘often’ (56%, n = 102); ‘sometimes’ (25%, n = 45) and ‘rarely’ or ‘never’ (15%, n = 26).

### 4.5. Conveyance to Hospital

Respondents identified several factors influencing the decision to convey patients at end of life to hospital. General Practitioner (GP) recommendation was the most common reason for conveyance to hospital with 80% (n = 146) of respondents stating this was ‘always’ or ‘often’ the reason. The second most common reason was family request. Overall, 64% (n = 118) of respondents stated that this was ‘always’ or ‘often’ the reason and a further 30% (n = 54) stated that this was ‘sometimes’ the reason.

Patient care needs and requests from family members were frequently reported as reasons for hospital conveyance. Symptom control management (e.g., pain, nausea/vomiting, urinary retention, ascitic drainage), was the third most common reason for conveyance to hospital with 60% (n = 110) of respondents stating this was ‘always’ or ‘often’ the reason and 33% (n = 60) ‘sometimes’ the reason. Recommendation from another healthcare professional was the next most common reason with 49% (n = 88) stating ‘always’ or ‘often’ and 36% (n = 64) indicating ‘sometimes’. Palliative care emergencies, including spinal cord compression, hypercalcaemia, haemorrhage, neutropenic sepsis and superior vena cava obstruction, were reported as a reason for conveyance ‘always’ or ‘often’ by 44% (n = 81) and ‘sometimes’ by 28% (n = 51).

### 4.6. Availability of Referral Pathways and Access to Advice

Respondents reported variation in their ability to access referral pathways for patients in the last year of life, both during and outside normal working hours (Table 3). In-hours, 51.1% (n = 93) could ‘often’ or ‘always’ contact/refer to the GP, 35.7% (n = 65) could contact/refer to the community nursing team in-hours and 32.4% (n = 59) could contact/refer to specialist palliative care teams in-hours. Access to some other services was more limited. Almost two-thirds (62.6%, n = 114) reported that they could ‘rarely’ or ‘never’ contact or refer to oncology teams in-hours, increasing to 79.1% (n = 144) out-of-hours. In addition to referral pathways, respondents reported difficulties accessing professional advice and specialist palliative care support. Lack of availability of healthcare professionals/teams for advice was reported ‘often’ or ‘always’ both in and out-of-hours (see Table 4). Furthermore, 62.6% (n = 114) reported a lack of specialist palliative care team (hospice) input ‘always’ or ‘often’.

### 4.7. Challenges Encountered

Respondents identified challenges across several areas of end-of-life care, including access to patient information, confidence and competence, community service support, conflicting views regarding care decisions, and education and training.

Access to information: Limited access to patient information was commonly reported. Overall, 64% (n = 117) reported they ‘always’ or ‘often’ lacked patient medical history; 28% (n = 51) ‘sometimes’ lacked access to medical history. Access to Advance Care Planning information was also limited, with 89% (n = 162) reporting that they ‘always’ or ‘often’ lacked access to Advance Care Planning discussions and 88% (n = 160) reporting that they ‘always’ or ‘often’ lacked access to existing Advance Care Planning documentation.

Fear, confidence, and competency: Respondents also reported challenges relating to decision-making and their perceived confidence and competence in providing end-of-life care. Almost half (47%, n = 85) reported experiencing a ‘fear of doing the wrong thing’ ‘always’ or ‘often’; 40% (n = 72) ‘sometimes’ and 14% (n = 25) ‘rarely’ or ‘never’. In relation to ‘not feeling confident with medicines,’ 30% (n = 56) indicated that they felt like this attending patients at end-of-life; 39% (n = 71) ‘sometimes’ and 30% (n = 54) ‘rarely’ or ‘never’.

**Figure 2:**
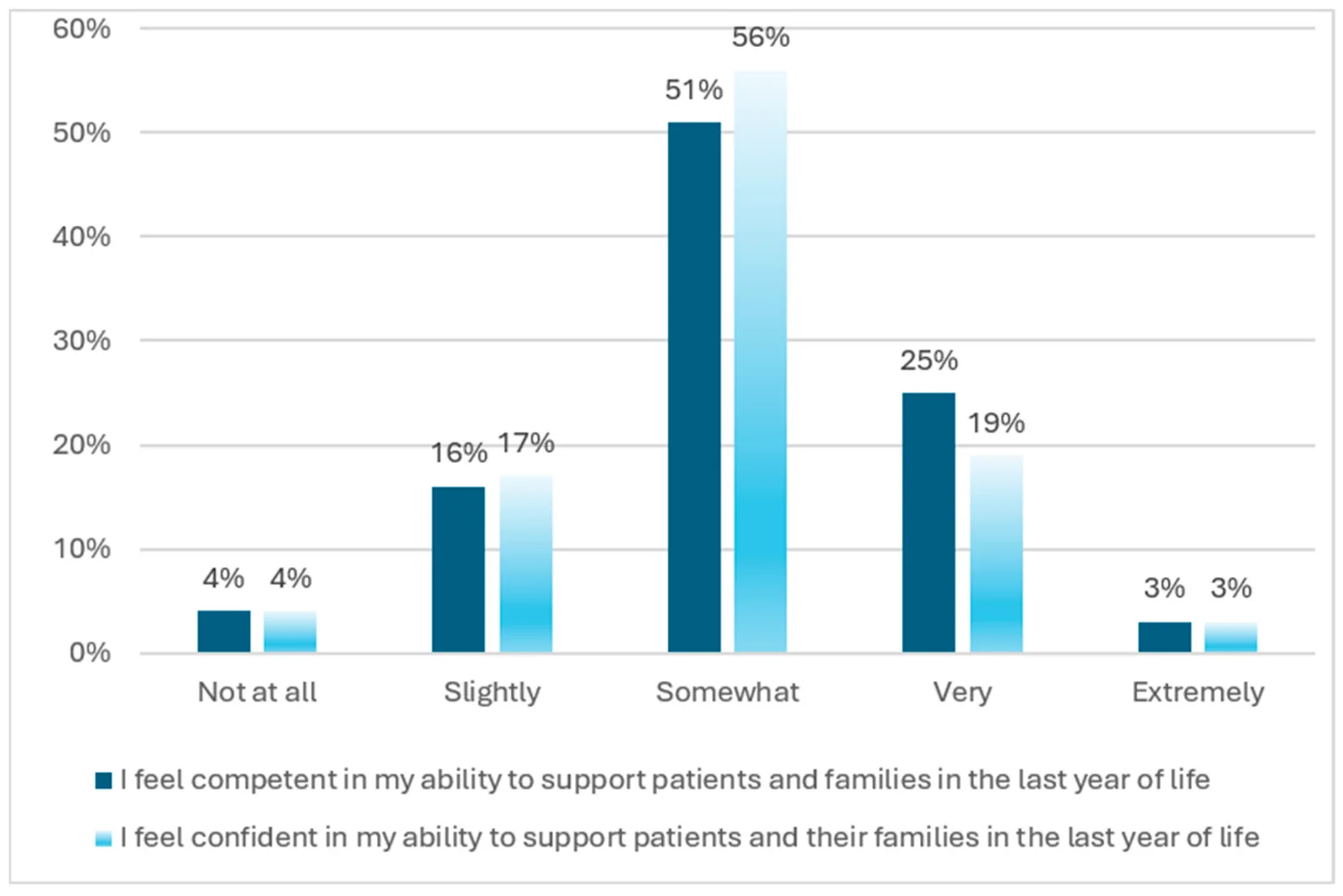
How competent and confident do you feel in your ability to support patients and families in the last year of life?

In relation to the statement ‘I feel competent in my ability to support patients and families in the last year of life,’ 4% (n = 8) stated ‘not at all’, 16% (n = 30) ‘slightly’, 51% (n = 93) ‘somewhat’, 25% (n = 45) ‘very’ and 3% (n = 6) ‘extremely’. Responses to the statement ‘I feel confident in my ability to support patients and families in the last year of life,’ showed that 4% (n = 8) responded ‘not at all’, 17% (n = 31) ‘slightly’, 56% (n = 101) ‘somewhat’, 19% (n = 35) ‘very’ and 3% (n = 6) ‘extremely’ (Figure 2).

Community Service Pressures: Challenges were also reported in relation to the availability of community-based support. Overall, 76% (n = 137) reported ‘always’ or ‘often’ having insufficient GP service support; 20% (n = 36) ‘sometimes’ and 5% (n = 9) ‘rarely’ or ‘never’. In total, 64% (n = 117) stated that insufficient community nursing team support was ‘always’ or ‘often’ a challenge; 26% (n = 48) ‘sometimes’ and 9% (n = 17) ‘rarely’ or ‘never’.

Conflicting Views: Respondents reported challenges arising from differing views about patient care and conveyance. Difficulties challenging conveyance recommendations made by other healthcare professionals were reported ‘always’ or ‘often’ by 59% (n = 106), ‘sometimes’ by 31% (n = 57) and ‘rarely’ or ‘never’ by 10% (n = 18). Conflicting views between different parties (crew, family, GP) was reported as ‘always’ or ‘often’ a challenge by 58% (n = 106). Twenty-seven percent (n = 50) felt it was ‘sometimes’ a challenge and 14% (n = 26) ‘rarely’ or ‘never’ a challenge. With regard to family conflict and/or conflict between patient and family, 27% (n = 50) felt that this was ‘always’ or ‘often’ a challenge; 46% (n = 83) ‘sometimes’ and 27% (n = 49) ‘rarely’ or ‘never’ a challenge.

Education and Training: Respondents reported challenges associated with their preparation for providing end-of-life care. Overall, 39% (n = 71) indicated ‘always’ or ‘often’; 41% (n = 74) ‘sometimes’ and 20% (n = 37) ‘rarely’ or ‘never’. Lack of end-of-life care education was reported at both pre- and post-registration levels. Sixty-seven percent (n = 121) stated ‘always’ and ‘often’; 27% (n = 49) ‘sometimes’ and 6% (n = 11) ‘rarely’ or ‘never’. In terms of the lack of end-of-life care training/education (continuing professional development) post-registration influenced respondents’ ability to meet the needs of their patients, 71% (n = 129) stated ‘always’ or ‘often’, 24% (n = 43) ‘sometimes’ and 5% (n = 9) ‘rarely’ or ‘never’. Thirty-seven percent (n = 67) stated that they had undertaken continuing professional development end-of-life care training/education specifically for paramedics and 40% (n = 72) had undertaken continuing professional development end-of-life training/education for healthcare professionals not specifically for paramedics.

## 5. Discussion

Findings provided insight into the perceptions and experiences of paramedics delivering end-of-life care, showing that paramedics frequently attend patients at end of life but do so in settings characterised by limited access to patient information, inconsistent referral pathways and a lack of confidence linked to insufficient training. These challenges are similar to those reported in Campling et al.’s study in England [11]. Their occurrence within Northern Ireland, despite its distinct regional and integrated Health and Social Care structure, demonstrates that several challenges previously identified are also reported by paramedics in the Northern Ireland context.

The findings mirror those of Campling et al., in which 81% of respondents reported lacking access to Advance Care Planning discussions and 77% reported absence of Advance Care Planning documentation [11]. Similarly, in the present survey, 89% and 88% of respondents reported lacking access to Advance Care Planning discussions and documentation, respectively. These findings indicate that limited access to end-of-life care information is commonly perceived by paramedics as a challenge in practice. The similarity between findings from Northern Ireland and those from 11 English ambulance services suggests that difficulties accessing end-of-life care information may reflect a wider challenge across ambulance services rather than being specific to a single regional service.

The present study did not examine whether lack of information directly influenced hospital conveyance decisions or patient outcomes. However, previous research has reported that limited access to relevant patient information may contribute to more risk-averse decision-making and hospital conveyance, including where this may not align with patient or family preferences [7,10]. Other international work has also suggested that improved access to digital care information and anticipatory prescribing arrangements may support community-based end-of-life care and reduce potentially avoidable conveyance [16]. These findings from the wider literature provide context for the information-access challenges reported by respondents in the present study.

In both this survey and Campling et al.’s study, respondents reported limited confidence and perceived competence, with inadequate pre- and post-registration education identified as a contributing factor [11]. Confidence and competence were self-reported and not objective assessments of clinical competence or professional performance.

Findings from this survey indicated that respondents commonly perceived education and training as influencing their ability to meet patients’ end-of-life care needs, with 67% reporting insufficient pre-registration preparation and 71% reporting insufficient post-registration training. This supports international calls for curriculum reform and development of paramedic competencies regarding palliative care [15,16]. However, training alone is unlikely to be sufficient without corresponding service infrastructure and pathways that allow paramedics to act on their training and confidently make community-informed decisions.

‘Fear of doing the wrong thing’ emerged as a challenge in both this survey and Campling et al.’s, reflecting ongoing tension between emergency care culture and palliative care principles [11]. Previous research has described moral distress and emotional labour among paramedics when navigating the transition between a traditional life-preservation approach and a palliative approach to care [14,20]. Further research exploring the emotional impact of providing end-of-life care on paramedics may be warranted.

Findings should be considered within the wider organisational and service context in which paramedics provide end-of-life care. Although this study did not directly examine organisational culture, resource allocation or workforce pressures, several findings suggest that challenges extend beyond individual paramedic knowledge and confidence. Reported difficulties accessing patient information, medicines, referral pathways, community services, and specialist palliative care support indicate that organisational and system-level factors may influence paramedics’ ability to provide end-of-life care. The reported tension between the life-preservation focus of paramedic training and the ethos of end-of-life care may reflect the continuing influence of the traditional emergency care focus of ambulance services. These findings suggest that education and training should be considered alongside access to patient information and medicines, referral pathways, community and specialist palliative care support, and the wider service infrastructure required to support paramedics’ decision-making and end-of-life care delivery in the community.

## 6. Policy and Practice Implications

The findings identify several areas that may warrant consideration in future policy and service development to support paramedics providing end-of-life care.

System-level considerations: One potential area for service development is improved interoperability between ambulance services and wider health and social care information systems, enabling paramedics to access relevant Advance Care Planning documentation at the point of care. The reported difficulties accessing patient information highlight the importance of ensuring that paramedics have access to documented patient preferences and treatment plans when making end-of-life care decisions. The reported difficulties accessing medicines required for symptom control also indicate that medication access should be considered in future service development.

Workforce development: There are workforce development implications in relation to the identified need for end-of-life education at both pre- and post-registration levels. Embedding end-of-life care competencies into paramedic training programmes, supported by continuing professional development pathways, may help to build knowledge and confidence in providing end-of-life care.

Care pathways and specialist support: Accessible palliative care advice and improved referral pathways may provide additional support for paramedics’ making decisions about care and conveyance in the community. Accessible palliative care advice lines, tailored for paramedic use, could provide real-time support during decision-making. Improving access to appropriate referral pathways, including community nursing, oncology and specialist palliative care services, may provide additional support for paramedics delivering end-of-life care in the community.

The impact of these potential service developments on paramedic decision-making, hospital conveyance, patient experiences and end-of-life care outcomes would require further evaluation.

## 7. Limitations

A census approach was used, with all eligible Northern Ireland Ambulance Service paramedics invited to participate; however, the 34% response rate introduces the potential for non-response bias. It is possible that paramedics who chose to participate had greater experience of, interest in, or stronger views about end-of-life care than those who did not respond. Paramedics experiencing greater workload or service pressures may have been less able to participate. The online mode of participation may also have influenced who chose to respond, although the extent and direction of any resulting selection bias cannot be determined. Although respondent age demographics were broadly comparable with Northern Ireland Ambulance Service workforce data, comparable workforce data using the questionnaire role categories was not possible as survey respondents could select multiple roles, reflecting the nature of their practice. Findings should therefore be interpreted as reflecting the experiences and perceptions of respondents and should not be assumed to represent those of all Northern Ireland Ambulance Service paramedics.

Given the differences in ambulance service organisation, scope of practice, palliative care provision, and access to community services across healthcare systems, the findings should not be transferrable beyond the Northern Ireland context. However, they may offer insights for ambulance services operating within comparable organisational and service contexts. Further multi-centre research across different ambulance service and healthcare settings is required to determine the extent to which these findings are shared across contexts.

Data were self-reported and may be subject to recall or social desirability bias. The survey measured the respondents’ perceived competence and confidence specifically in supporting patients and families in the last year of life; however, it was not designed as an objective assessment of clinical competence or professional standards. The study did not collect objective clinical data on actual hospital conveyance or patient outcomes. The descriptive cross-sectional design limits causal inference.

Although internal consistency of relevant domains of the adapted ParAid Questionnaire was assessed, with Cronbach’s alpha coefficients ranging from 0.725 to 0.895, no additional formal assessment of construct or criterion validity was undertaken as part of the present study. This should be considered when interpreting findings obtained using the adapted questionnaire.

## 8. Conclusions

Paramedics deliver end-of-life care in complex, high pressure environments with limited support and inconsistent access to patient information. Respondents identified gaps in education, limited access to documentation, and challenges with referral pathways as factors that may affect their confidence and ability to provide home-based end-of-life care that takes account of patient preferences. The findings identify information accessibility, specialist clinical support, integrated referral pathways, and palliative care education as potential areas for future service development. Strengthening these systems may better support paramedics in delivering end-of-life care and making decisions about appropriate care and conveyance.

## Figures and Tables

**Table 1:** Respondent Characteristics.

Age (Years)	% (n)
21–25	6 (11)
26–30	8 (15)
31–35	19 (35)
36–40	15 (28)
41–45	14 (26)
46–50	14 (26)
51–55	10 (19)
56–60	9 (17)
61–65	3 (5)
Number of years registered as a paramedic	% (n)
5 or under	39 (71)
6–10	16 (29)
11–15	15 (27)
16–20	13 (24)
21–25	10 (18)
Over 26	7 (13)
Role	% (n)
Frontline clinical operations paramedic	88 (160)
Specialist/advanced operational role (extended scope of practice)	9 (17)
Leadership role	27 (49)
Other *	8 (15)

* Other role includes Integrated Clinical Hub, Research, Education/Training, Control Room Clinical Support, Bank Paramedic. Notes: Percentages may not add up to exactly 100% due to rounding. Respondents could select more than one role; therefore, frequencies and percentages for role categories are not mutually exclusive.

**Table 2:** Frequency of actions when attending patients in the last year of life.

	Always % (n)	Often % (n)	Sometimes % (n)	Rarely % (n)	Never % (n)	No Response % (n)
Provide support and advice to family and friends	41.2 (75)	48.4 (88)	8.8 (16)	1.1 (2)	0 (0)	0.5 (1)
Provide support and advice to care/nursing home staff	17 (31)	43.4 (79)	28.6 (52)	9.9 (18)	1 (1)	0.5 (1)
Administer medicines for symptom control	3.8 (7)	29.7 (54)	47.3 (86)	16.5 (30)	2.2 (4)	0.5 (1)
Convey the patient to hospital (emergency department)	3.3 (6)	39 (71)	45.6 (83)	8.8 (16)	1.6 (3)	1.6 (3)
Convey the patient to a hospice	0 (0)	1.1 (2)	5.5 (10)	42.3 (77)	50 (91)	1.1 (2)
Refer to other healthcare providers for ongoing patient care	7.1 (13)	50 (91)	35.7 (65)	4.9 (9)	1.6 (3)	0.5 (1)
Support arrangements for care after death	6.6 (12)	22.5 (41)	25.3 (46)	25.8 (47)	18.1 (33)	1.6 (3)

Note: Percentages are calculated using the total study sample (n = 182). ‘No response’ represents missing responses for each item. Percentages may not sum to exactly 100% due to rounding.

**Table 3:** Which of the following referral pathways are you able to access for patients in their last year of life?

		Often or Always % (n)	Sometimes % (n)	Rarely or Never % (n)	Not Applicable % (n)	No Response % (n)
General Practitioner	In-hours Out-of-hours	51.1 (93) 61.5 (112)	38.5 (70) 23.1 (42)	9.9 (18) 14.8 (27)	0.5 (1) 0.5 (1)	0 (0) 0 (0)
Community Nursing Team	In-hours Out-of-hours	35.7 (65) 9.9 (18)	42.3 (77) 28.6 (52)	20.9 (38) 59.9 (109)	0.5 (1) 0.5 (1)	0.5 (1) 1.1 (2)
Specialist Palliative Care Team (hospice)	In-hours Out-of-hours	32.4 (59) 11.5 (21)	36.8 (67) 24.2 (44)	29.1 (53) 61.5 (112)	0.5 (1) 1.6 (3)	1.1 (2) 1.1 (2)
Oncology Team	In-hours Out-of-hours	13.7 (25) 4.4 (8)	19.2 (35) 12.6 (23)	62.6 (114) 79.1 (144)	4.4 (8) 3.3 (6)	0 (0) 0.5 (1)
Rapid Response Team	In-hours Out-of-hours	9.9 (18) 3.8 (7)	17.6 (32) 10.4 (19)	66.5 (121) 79.7 (145)	6 (11) 4.9 (9)	0 (0) 1.1 (2)
Urgent Community Response Team	In-hours Out-of-hours	7.1 (13) 2.7 (5)	20.3 (37) 10.4 (19)	64.8 (118) 79.7 (145)	7.7 (14) 5.5 (10)	0 (0) 1.6 (3)

Note: Percentages are calculated using the total study sample (n = 182). Percentages may not add up to exactly 100% due to rounding.

**Table 4:** Routes to Advice and Referral.

		Often or Always % (n)	Sometimes % (n)	Never or Rarely % (n)	No Response % (n)
Lack of availability of healthcare professionals/teams for advice	In-hours Out-of-hours	55.5 (101) 80.2 (146)	33.0 (60) 14.3 (26)	11.5 (21) 5.5 (10)	0 (0) 0.5 (1)
Lack of availability of healthcare professionals/teams for immediate referral/hand over	In-hours Out-of-hours	64.8 (118) 82.4 (150)	22.5 (41) 12.6 (23)	12.6 (23) 3.8 (7)	0 (0) 2 (1.1)
Lack of specialist palliative care team (hospice) input		62.6 (114)	26.9 (49)	10.4 (19)	0 (0)

Note: Percentages are calculated using the total study sample (n = 182). Percentages may not add up to exactly 100% due to rounding.

## Data Availability

Anonymous participant data may be made available from the lead author upon reasonable request as privacy concerns.
